# Not explicit but implicit memory is influenced by individual perception style

**DOI:** 10.1371/journal.pone.0191654

**Published:** 2018-01-25

**Authors:** Kyoko Hine, Yoshiaki Tsushima

**Affiliations:** 1 Department of Information Environment, Tokyo Denki University, Inzai-shi, Chiba, Japan; 2 Center for Information and Neural Networks, National Institute of Information and Communications Technology and Osaka University, Osaka, Japan; 3 Sutokuin Lab, Osaka, Japan; Waseda University, JAPAN

## Abstract

Not only explicit but also implicit memory has considerable influence on our daily life. However, it is still unclear whether explicit and implicit memories are sensitive to individual differences. Here, we investigated how individual perception style (global or local) correlates with implicit and explicit memory. As a result, we found that not explicit but implicit memory was affected by the perception style: local perception style people more greatly used implicit memory than global perception style people. These results help us to make the new effective application adapting to individual perception style and understand some clinical symptoms such as autistic spectrum disorder. Furthermore, this finding might give us new insight of memory involving consciousness and unconsciousness as well as relationship between implicit/explicit memory and individual perception style.

## Introduction

Our physical and mental activities are crucially influenced by the past experiences recorded in memory. Generally, there are two types of memories, explicit and implicit memories. Explicit memory refers to the memory that involves conscious recollection of information, on the other hand, implicit memory does not depend on conscious recollection [1–3]. Not only explicit but also implicit memory has considerable influence on our physical and mental activities. For example, we plan a summer vacation with and/or without conscious sight of travel advertisements. However, it is not clear whether both explicit and implicit memory vary among different individuals.

**Fig 1:**
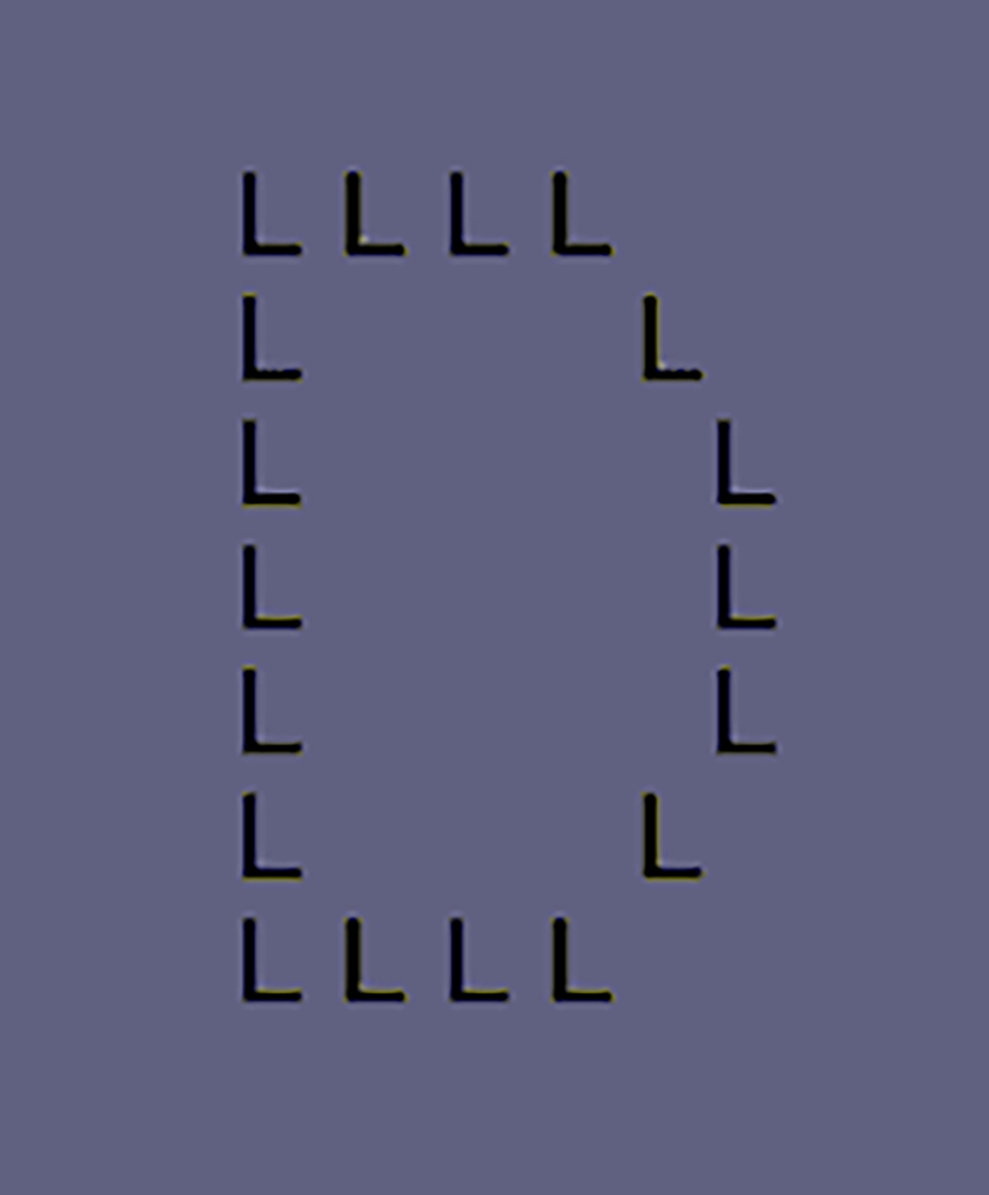
An example of a Navon figure. “D” consists of “L”s. When globally seeing it, we can read it as “D”. When locally seeing it, we will read a lot of “L”s.

It is well known that our perception styles are different with each person. For instance, Bouvet et al. used a Navon figure [4], a large letter consisting of small letters (Fig 1), and found that the individual perceptual bias with which global properties (reading a large letter) tend to be perceived is not equally found across individuals [5]. They concluded that there are individual differences in global or local perception style. Do such different perception styles affect explicit and/or implicit memory?

Psychologists suggest that memory involves three essential aspects of information processing, encoding, storage, and retrieval [6]. Encoding is defined as input processing of information; storage is keeping information over time; retrieval refers to the ability to access stored information. Encoding is the first process of forming memory, and perception is one part of encoding information into memory. Therefore, individual perception style might be related to explicit and/or implicit memory in some way. However, this prediction has not been systematically explored yet.

**Fig 2:**
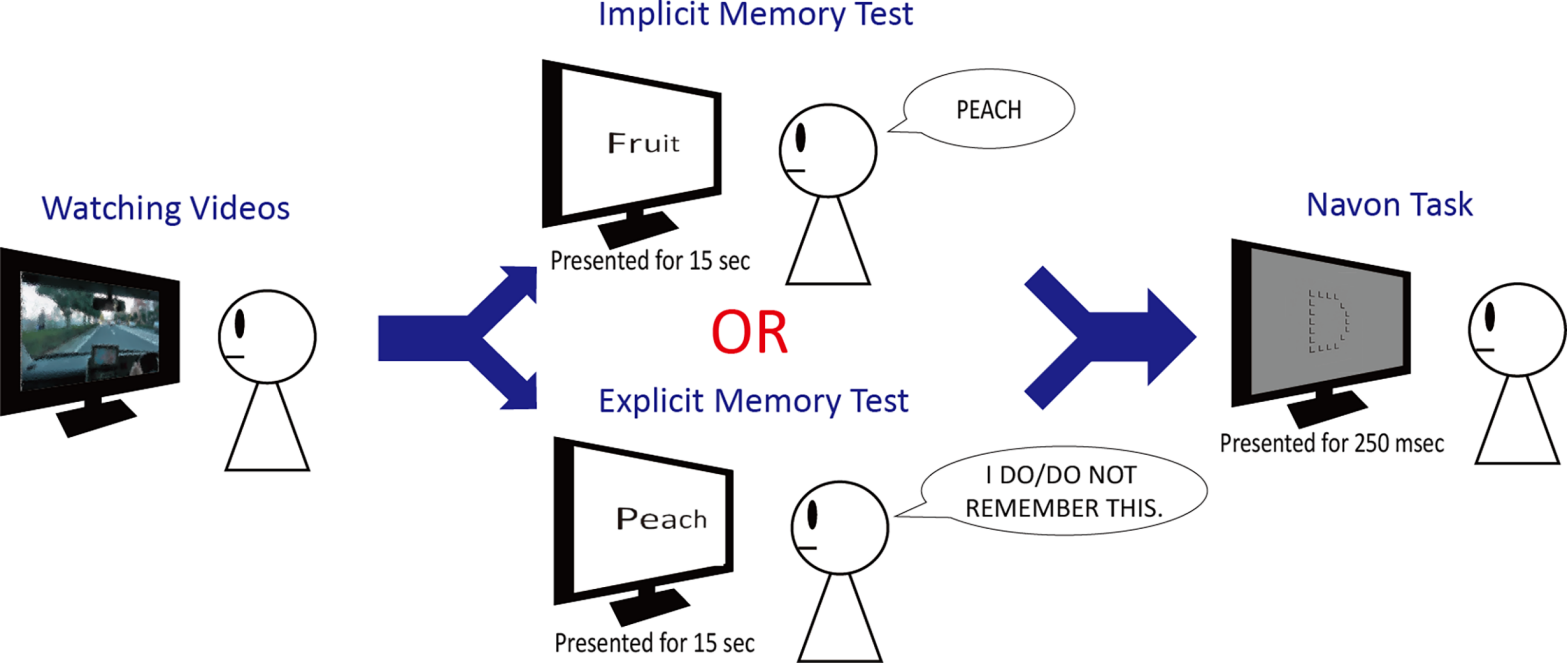
Experimental design. After participants viewed 17 short videos, they completed either an implicit or explicit memory test. After the memory test, all of them took the Navon task to check their individual perception style.

Here, we examined how individual perception style (global or local) correlates with implicit and explicit memory. To investigate that, we conducted a series of psychological experiments, with two types of memory tests and a Navon task (Fig 2).

We first did the memory test for the estimation of implicit or explicit memory, in which participants viewed the video consisting of 17 short videos and answered the questions. In the implicit memory test, we used a free association task for estimating the status of implicit memory: participants were asked to answer an associable word to given words [7–12]. In the explicit memory test, participants were asked to report whether the given words were presented as words/objects in the video. After the memory test, for the assessment of individual perception style (relatively global or local), we used Navon task in which participants read large alphabet (global condition) or small letters (local condition) in a large alphabet consisting of small letters (Fig 1) [4,5,13,14].

## Materials and methods

### Procedures

#### Implicit memory test

Participants viewed 17 videos of scenery from a car window: We shot those videos in the city streets of Kanagawa prefecture Japan. The average of video length was 31 ± 4.3 secs. Participants were not told that they would take a memory test after watching those videos. The 17 videos were randomly and continuously run for each participant (It took total almost 11 minutes.). After viewing those videos, they took the implicit memory test in which participants were asked to tell about an associated word to the given words (e.g. They answered “Peach” to <Fruit>) with their writings in Japanese [7–12]. We created the 30 given words associated with words/objects presented in the videos. From preliminary experiments, we confirmed that we could identify those words/objects in the videos. These given words were carefully selected with consideration to “category norm” issue. Category norm is the first word coming to mind to an association word [15–19]. They generally depend on each person’s background such as culture and/or generation [16] (e.g. “Apple” firstly comes to the Japanese young’s mind to “Fruit”.) (S1 Table). To ensure that we could distinguish participants’ answers from category norms or words/objects presented in the videos, we set the given words according to the rule that the words/objects presented in the videos (Target answer) did not belong to the category norms. For example, we did not set “male accessory” as the given words, because the Target answer (“necktie” presented in the video) is the category norm to “male accessory” [17]. In addition, we set other thirty given words that were related to words/objects non-presented in the videos to prevent the possibility that participants noticed some associations between the given words and the contents of videos (Filler items). Therefore, the total number of given words in the implicit test were sixty. The order of the 60 given words was counterbalanced across participants. In the memory test, each word was presented at the monitor for 15 secs (Fig 2) based on previous research [7–12].

In order to check the possibility that participant’s answers were from their explicit memory, all participants were asked whether or not they were aware of some sorts of association between the given words and the contents of the videos at the end of the all experiment [20]. Also, we asked them whether they knew the streets in the videos to check their familiarity with them.

#### Explicit memory test

Participants viewed 17 videos used in the implicit memory experiment, and the experimental procedures were the same as the implicit memory experiment, except that they took the explicit memory test in which participants were asked whether they recognized the words as words/objects in the videos (e.g. They answered “I do/do not remember this” to <Peach>). Thirty given words were presented words/objects in the videos, and those words were chosen based on the Target answers in the implicit test, because we would like to assess the same words/objects of implicit and explicit memory as much as possible [21,22] (also see S1 and S2 Tables). In addition, we set other thirty given words that were not presented in the videos to get more accurate memory performance of the explicit memory test (Foil items) [23, 24]. Therefore, the total number of given words in the explicit test were sixty. The order of the 60 given words was counterbalanced across participants. In the test, each word was presented at the monitor for 15 secs (Fig 2) based on previous research [7–12]. In the same manner of the implicit memory test, we asked them whether they knew the streets in the videos to check their familiarity with them.

#### Navon task

Each trial began with a presentation of “Big” or “Small” in Japanese for 1000 msecs. Then, the fixation cross was presented at the center of the screen for 1000 msecs. After the blank for 1000 msecs, a Navon figure was presented for 250 msecs. When “Big” was presented at the beginning of the trial (global condition), participants were required to answer the large letter in the Navon figure from the three options (e.g. They selected “D” from three letters such as “D”, “S”, and “L” in the case of Fig 1.). When “Small” was presented (local condition), participants had to answer the small letter (e.g. The correct answer is “L.” in the case of Fig 1.). Participants were asked to respond as quickly as possible. The inter trial interval (ITI) was 1000 msecs. The order of global or local condition was randomized and counterbalanced, because the biased order of Navon task alters the performance [25, 26]. The total number of trials was 72 trials, 36 for the global condition and other half for the local condition.

#### Analyses

To evaluate the performance of the implicit and explicit memory tests, we used the following analytical calculation methods. The performance of the implicit memory test was calculated on that the number of Target answer was divided by thirty (the total number of 30 given words associated with the words/objects presented in the videos) (S1 Table), because we could ignore the answers to 30 Filler items (as described before). On this occasion, we carefully graded their scores to prevent counting the category norms [15–19] as their response words from implicit memory (S1 Table). The performance of the explicit memory test was calculated on that the number of correct answers was divided by sixty (the total number of given words in the explicit memory test), because we should deal with both presented and non-presented items to more accurately evaluate the explicit memory (as described before). Additionally, for comparably analyzing and viewing the performance of the implicit memory test and the explicit memory test, the performance scores of the implicit and explicit memory test were adapted Z-Transformation. To assess the individual perception style, the index of perception style was calculated on that the number of errors of Navon task in local condition was subtracted from the number of errors in global condition. Therefore, the positive value of the index of perception style indicated that the fewer errors in local condition in comparison with that in global condition.

### Participants

We calculated the sample size using G*Powers [27] based on some previous studies that had very similar topic and experimental design and parameter [5, 28]. We set a bivariate normal model with a two tail test, α = 0.05, and 0.80 power. The power analysis indicated 18 participants per group allowed for the examination of the correlation of the memory performance and the perception style. From that result, we assigned 18 participants to each experiment group.

Eighteen participants (5 females and 13 males; aged from 18 to 23; mean age = 19.9) were assigned to the implicit memory experiment group. Another eighteen participants (6 females and 12 males; aged from 18 to 26; mean age = 21.0) took the explicit memory experiment. Participants were randomly assigned to the explicit or implicit memory tests. All participants signed the letter of consent approved by the Ethics Committee of the Tokyo Denki University in compliance with Declaration of Helsinki.

## Results

First of all, from results of the Navon task, we found 4 participants who had 0 for their perception style scores (3 in implicit group and 1 in explicit group.). Therefore, we checked the detailed performance of the Navon task about those four participants. The results show that the mean of the reaction time for each participant was within one standard deviation of the average for each group. In other words, those participants were not significantly slow in responding to ensure accuracy and there was not a speed-accuracy tradeoff. Thus, we concluded that 0 index represents just lack of a strong perceptual bias [5]. On top of that, the brief questionnaire at the end of experiments found that no participants in both implicit and explicit memory tests were familiar with streets in the videos. Also, participants in the implicit memory experiment group did not report their awareness of the association between the given words and the contents of the video, therefore, we decided that we can use all data for analysis of the implicit memory [20].

**Fig 3:**
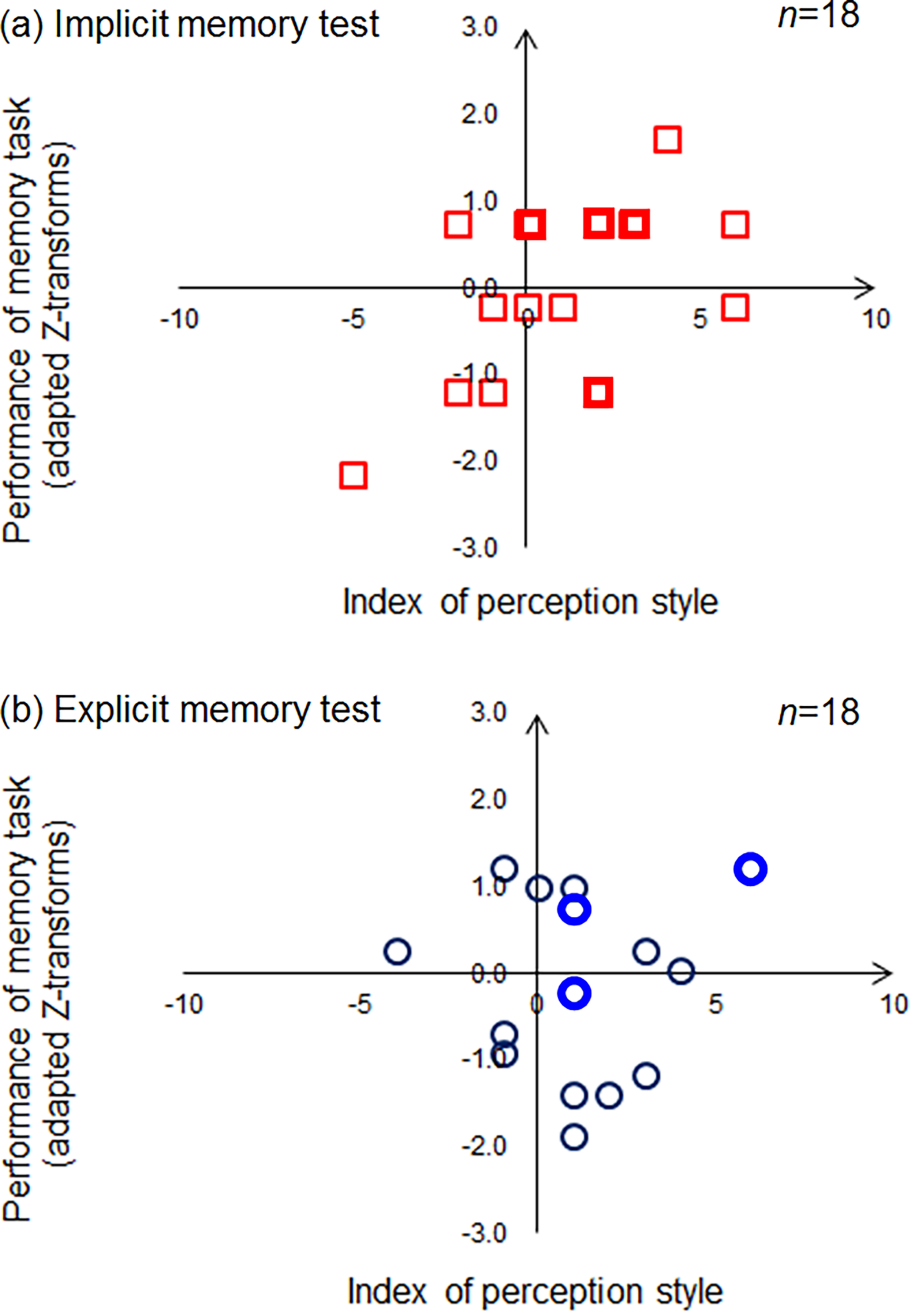
Scatter graphs of the performance of the memory tests as a function of the index of perception style. Bold plots represent overlaps of two participants. (a) Scatter graph of the performance of the implicit memory test as a function of the index of perception style (b) Scatter graph of the performance of the explicit memory test as a function of the index of perception style.

Fig 3 represents two scatter graphs of the performance of the memory tests as a function of the index of perception style, implicit memory test with the perception style (Fig 3(A)) and explicit memory test with the perception style (Fig 3(B)).

As a result, significant correlation was found between the performance of the implicit memory test and the index of perception style (Table 1). The performance of the implicit memory test increases as strength of the bias of local perception. On the other hand, we could not find any correlation between the performance of the explicit memory test and the index of perception style (Table 1).

**Table 1:** Results of the correlated t-tests.

	df	*r*	*p*-value
Implicit	18	0.52	0.04*
Explicit	18	0.18	0.49

*p<0.05.

The correlation between the performance of the implicit memory test and the index of perception styles was significant, whereas the correlation between the performance of the explicit memory test and the index of perception styles was not significant.

In case that including the responses for Foil items (thirty given words that were not presented in the videos) for analysis made the current results of explicit memory test, we also analyzed the responses for only presented words/objects in the videos and calculated the correlation between the performance and the index of perception style. As a result, we did not find any correlation between them (*r* = 0.10, *p* = 0.42). Therefore, containing the responses for Foil items into the performance of the explicit memory test did not crucially affect the present main findings. Additionally, one might think that we should use A-prime values for evaluating results of the explicit memory test because of dealing with the response bias [29]. So, we calculated the correlation between the A-prime values and the index of the perception style. The results showed the same tendency as Table 1 and Fig 3B (*r* = 0.21, *p* = 0.39, also see S1 Fig). In the current experiment, we therefore concluded that the response bias of the explicit memory test did not essentially affect the present main findings.

## Discussion

In the current research, we examined whether implicit and explicit memories varied between individual perception styles. The obtained results demonstrate two significant things. Firstly, not explicit but implicit memory is influenced by individual perception style. This indicates that our individual sensory differences have some effects on unconscious processing rather than conscious processing [1–3]. If this is the case, personal characteristics including perception style would correlate with deep inside the mind, such as the part of iceberg hidden under the sea [30]. In addition, explicit memory uninfluenced by individual perception style might contribute to construct the collective memory that is shared among a group, such as history [31–33]. To check the possibility that participants’ tiredness affects the results of the Navon task (because they did it at the end), we conducted the analysis of variance for the number of error on the Navon task with the test type (implicit and explicit) and the error type (wrong level and non-presented level). Results did not show the main effect of the test type and the significant interaction between the test type and the error type of Navon task. Based on these results, the difference of participants’ tiredness between the implicit and explicit group did not significantly influence the main results in the present study (see S3 Table).

Secondly, local perception style people’s performance of the implicit memory test was higher than global perception style people’s. There is the possibility that the baseline for how good participants were at coming up with associated words related with their perception style. To test this, we calculated a correlation between the response rate of the category norm [16–18] about the non-presented items and their perception style. Consequently, we could not find the correlation between them (*r* = 0.29, *p* = 0.25, also see S2 Fig). Therefore, we concluded that local perception style people have a tendency of unconsciously encode exposed information. If such encoded information is accessible, local perception style people would make their summer vacation plans more based on a travel advertisement on the road on their ways to every day work than global perception style people, because such an advertisement on the road is generally perceived without consciousness. We would be better off designing an advertisement tailored to individual perception style [34, 35].

Furthermore, the present results might contribute to some clinical fields, such as knowing as much about autistic spectrum disorder symptoms. Previous research reported that autistic spectrum people showed distinctive interests in parts of objects rather than the whole [36, 37]. In other words, autistic spectrum people might have local perception style relatively. Therefore, taken together, autistic people’s thinking and action might be more related to their past experiences without consciousness. This point of view would help us to understand autistic spectrum disorder. Finally, this study might provide us with not only relationship between implicit/explicit memory and individual perception style, but also new intuition of memory involving consciousness and unconsciousness.

## Supporting information

S1 Fig Scatter graph of the A-prime of the explicit memory test as a function of the index of perception style. The mean A-prime was 0.79±0.04 (*n* = 18).
(TIF)

S2 Fig Scatter graph of the response rate of the category norm about non-presented items as a function of the index of perception style. (TIF)

S1 Table The list of the given words, target answers, and category norms in the implicit memory test. (TIF)

S2 Table The list of the given words and correct answers in the explicit memory test. (TIF)

S3 Table Results of the analysis of variance for the number of error on the Navon task with the test type (implicit, explicit) as between-subjects variable and the error type (wrong level, non-present level) as within-subjects variable. Wrong level error means that participants selected the large letter when in the local condition or the small letter when in the global condition. Non-present level error means that participants chose the letter that was not presented in the figure (e.g. “S” in the case of Fig 1.). The mean number of the wrong level error was significantly higher than that of the non-present level error.
(TIF)
