# Supplementary figures and images for: Not explicit but implicit memory is influenced by individual perception style
Source: PLoS One. 2018 Jan 25;13(1):e0191654. doi: 10.1371/journal.pone.0191654 (PMC5784939; doi:10.1371/journal.pone.0191654)

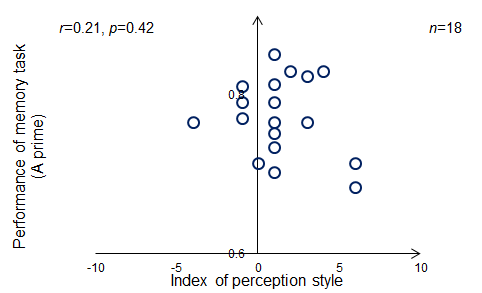

Supplement: S1 Fig — The mean A-prime was 0.79±0.04 (n = 18). (TIF) [file pone.0191654.s001.tif]

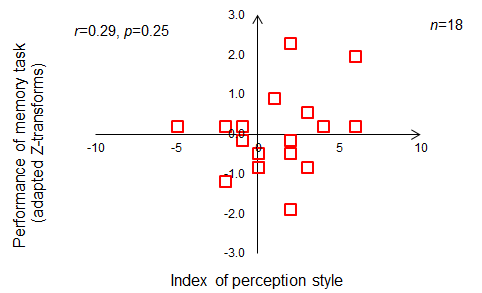

Supplement: S2 Fig — (TIF) [file pone.0191654.s002.tif]

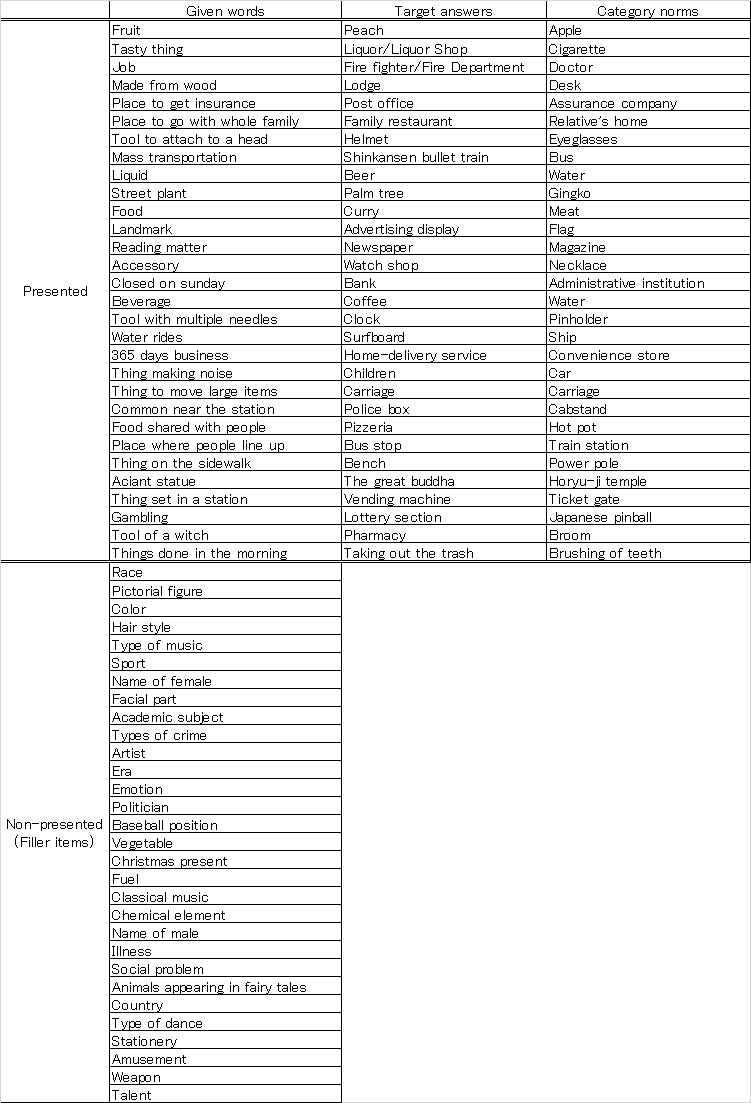

Supplement: S1 Table — (TIF) [file pone.0191654.s003.tif]

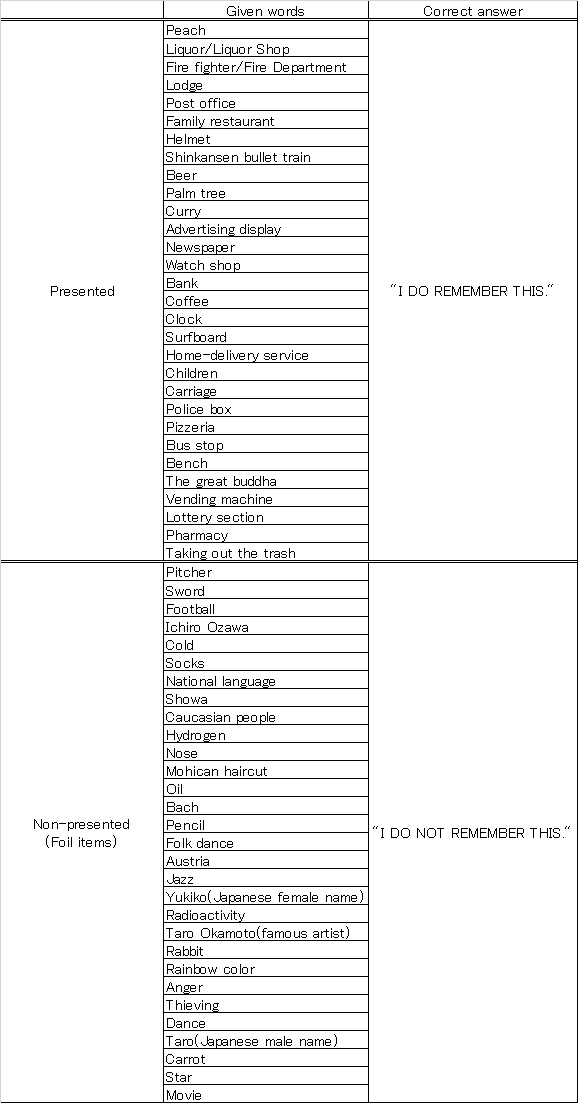

Supplement: S2 Table — (TIF) [file pone.0191654.s004.tif]

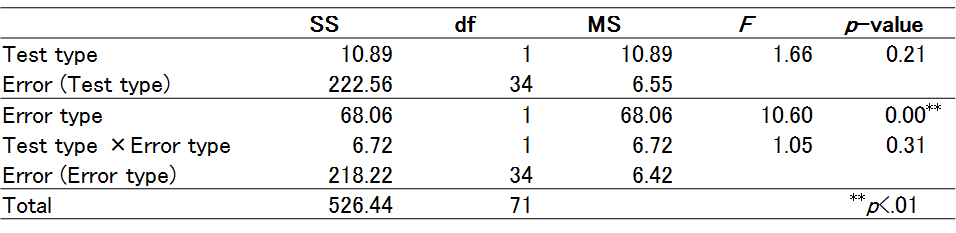

Supplement: S3 Table — Wrong level error means that participants selected the large letter when in the local condition or the small letter when in the global condition. Non-present level error means that participants chose the letter that was not presented in the figure (e.g. “S” in the case of Fig 1.). The mean number of the wrong level error was significantly higher than that of the non-present level error. (TIF) [file pone.0191654.s005.tif]
